# Hip and knee replacement in lower limb amputees: a scoping review

**DOI:** 10.1186/s12891-024-07342-z

**Published:** 2024-03-27

**Authors:** Thomas J. Walton, Abigail L. D. Chatterton, Victoria A. Goodwin

**Affiliations:** 1 NIHR Academic Clinical Fellow - Trauma and Orthopaedic Surgery, University of Exeter, St Lukes Campus, Magdalen Road, Exeter, Devon EX1 2LU UK; 2 University Hospitals Plymouth NHS Trust, Derriford Hospital, Plymouth, Devon PL6 8DH UK; 3 University of Exeter, St Lukes Campus, Magdalen Road, Exeter, Devon EX1 2LU UK

**Keywords:** Amputee, Total hip replacement, Total hip arthroplasty, Total knee replacement, Total knee arthroplasty, Hemiarthroplasty, Scoping review, Systematic review

## Abstract

**Background:**

There are many consequences of lower limb amputation, including altered biomechanics of gait. It has previously been shown that these can lead to increased rates of osteoarthritis (OA). A common and successful treatment for severe OA is joint replacement. However, it is unclear whether amputees undergoing this surgery can expect the same outcomes or complication profile compared with non-amputees. Furthermore, there are key technical challenges associated with hip or knee replacement in lower limb amputees. This scoping review aimed to identify and summarise the existing evidence base.

**Methods:**

This was a systematic scoping review performed according to PRISMA guidelines. An electronic database search of MEDLINE (PubMed), Cochrane Library, EMBASE and CINAHL was completed from the date of inception to 1^st^ April 2023. All peer reviewed literature related to hip or knee replacement among lower limb amputees was included.

**Results:**

Of the 931 records identified, 40 studies were included in this study. The available literature consisted primarily of case reports and case series, with generally low level of evidence. In total, there were 265 patients of which 195 received total hip replacement (THR), 51 received total knee replacement (TKR) and 21 received hip hemiarthroplasty. The most common reason for amputation was trauma (34.2%), and the main indication for joint replacement was OA (77.1%), occurring more frequently in the contralateral limb (66.7%). The outcomes reported varied widely between studies, with most suggesting good functional status post-operatively. A variety of technical tips were reported, primarily concerned with intra-operative control of the residual limb.

**Conclusion:**

There is a need for more observational studies to clearly define the association between amputation and subsequent need for joint replacement. Furthermore, comparative studies are needed to identify whether amputees can be expected to achieve similar functional outcomes after surgery, and if they are at higher risk of certain complications.

**Supplementary Information:**

The online version contains supplementary material available at 10.1186/s12891-024-07342-z.

## Background

In the UK, approximately 60,000 patients each year access specialised prosthetic services [1]. However, true estimates of the UK population living with amputation are difficult to make, with contributions from congenital limb deficiency, trauma, peripheral arterial disease and the military. The prevalence of new lower limb amputation is estimated as 26.3/100,000 among 50 to 84 year olds with peripheral arterial disease, amounting to over 25,000 amputations over a 6-year period alone [2]. Irrespective of the exact figure, there are a large number of people living with lower limb amputation, of which the majority will use prosthetics to walk. As with any ambulant individual, they are subject to the same risks of degenerative joint disease or traumatic injury that can affect non-amputees.

Modern prosthetics benefit from ongoing developments in materials science and production technology, coupled with an ever-improving understanding of fundamental gait biomechanics and stump-socket interface [3]. However, despite these ongoing advancements, a prosthesis will still alter the transfer of energy during the gait cycle compared with a normal biological limb, leading to increased contact forces on other joints through compensatory processes [4, 5]. This has been demonstrated through the increased rates of hip and knee osteoarthritis (OA) observed among lower limb amputees, particularly affecting the non-amputated limb [6–8]. Norwell et al. found a significantly increased prevalence of OA among amputees (16.1%) compared with non-amputees (11.7%), in a military population [8]. However, Welke et al. found no clear difference in prevalence from the general population, though amputees were noted to develop OA at an earlier stage, suggesting accelerated disease in pre-disposed individuals [9].

For people with symptomatic hip or knee osteoarthritis, undergoing joint replacement is a proven and effective treatment [10]. However, the presence of concomitant lower limb amputation poses a unique challenge to both the patient and surgeon. From the patient’s perspective, post-operative rehabilitation is made more challenging, as an amputated limb utilising a prosthetic may be unable to provide the same level of compensation to gait as a normal limb in non-amputees. Meanwhile, stump specific complications such as swelling and wound healing may also limit ability to weight bear, delaying ambulation and rehabilitation progress. From the surgical perspective, it is technically difficult to achieve the optimum length, alignment and rotation of an implant without a contralateral limb to reference. Furthermore, when operating on the amputated limb, the length of the residual limb may restrict manoeuvrability during surgical dissection and implantation, while limiting the range of suitable implants compared to non-amputees. Although joint replacement in amputees remains uncommon compared with the total population treated, understanding the optimum surgical management and likely effectiveness of joint replacement in these patients is important, as this can guide consent discussions regarding patient expectations and complication profile for a distinct population group, while providing surgeons with valuable insight for the intra-operative management of patients they will not treat frequently.

The primary aim of this study was to identify and summarise the existing evidence base relating to hip and knee replacement in lower limb amputees, to explore whether clinical outcomes and post-operative complications are comparable to non-amputees, and to synthesise reported intra-operative techniques.

## Methods

### Search strategy

An electronic search of MEDLINE (PubMed), Cochrane Library, EMBASE and CINAHL was completed (date of inception to 1^st^April 2023). The search terms used were developed through preliminary searches and included relevant MeSH terms. The final search strategy was refined in conjunction with an Information Specialist (see Supplementary appendices). ClinicalTrials.gov and Google Scholar index were searched for pre-print publications, and reference list searching of included studies was also performed. Non-peer reviewed articles were not explored. The protocol for this systematic scoping review was developed in conjunction with the Preferred Reporting Items for Systematic Reviews and Meta-analyses (PRISMA) statement for scoping reviews [11], and was registered prospectively with the Open Science Framework [12].

### Selection process

All articles identified by the search strategy were imported into Rayyan [13]. Relevant titles and abstracts were screened independently by two authors (TW and AC) and selected for full text retrieval, depending on conformity to eligibility criteria. This process was repeated following retrieval of relevant full text articles, to determine final inclusion. A third author (VG) was available to settle disputes if necessary.

### Eligibility criteria

Inclusion criteria were: (i) English language literature, (ii) Any original research or report; case series, case report, technical tip, (iii) Adults > 18 years, (iv) Undergoing hip or knee replacement, for any indication, (v) Concomitant pre-existing lower limb amputation, for any indication, (vi) Any outcome data. Specific exclusion criteria were also applied: (i) Conference abstracts or opinion pieces, (ii) Amputation after arthroplasty procedure.

### Data extraction

A data extraction form was developed, piloted and refined by TW and AC prior to expansion across the remaining included studies. Data on study characteristics, patient demographics, amputation (level, laterality, indication), operation (type, laterality, indication), outcomes, complications and technical tips were all collected and imported into Microsoft Excel (iOS Version 2.76).

### Quality assessment

A scoping review methodology without quality assessment was performed, according to the framework outlined by Arksey and O’Malley (2005) [14]. In the authors’ opinion, the nature of the studies available for review meant quality assessment would not meaningfully enhance interpretation or discussion of findings.

### Data synthesis

A descriptive and narrative data analysis was performed, to summarise the existing evidence.

## Results

**Fig. 1:**
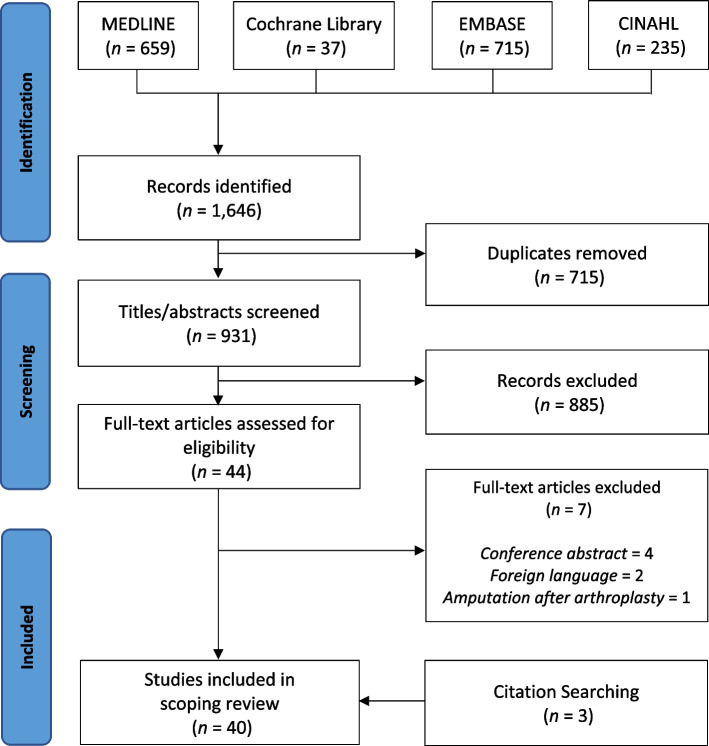
PRISMA diagram demonstrating search results and screening

Following screening, 40 studies were included in the review (Fig. 1). The majority of included studies were case reports or case series (Table 1) [15–54], from military and civilian populations across 15 different countries. The remainder included two case–control studies, a non-systematic review, and a technical article. There were no large-scale prospective cohort or comparative studies found, meaning the quality of available evidence did not exceed level IV.

**Table 1:** Summary of included studies

Author	Year	Location	Study Type	Total	Male	Female	Mean Age (years)
Amanatullah et al. [15]	2015	USA	Case series	35	25	10	60.7
Arango et al. [16]	2016	USA	Case report	1	1	0	62
Boussakri et al. [17]	2015	France	Case report	1	0	1	81
Cho et al. [18]	2018	South Korea	Case–control	54	54	0	67.8
Cho et al. [19]	2019	South Korea	Case–control	67	67	0	69.7
Christidis et al. [20]	2022	Greece	Case report	1	1	0	61
Constantin et al. [21]	2020	Australia	Case report	1	1	0	58
Crawford et al. [22]	2003	UK	Case report	1	0	1	75
Diamond et al. [23]	2013	UK	Case report	1	0	1	43
Dong et al. [24]	2022	USA	Case report	1	1	0	52
Dudhniwala et al. [25]	2011	UK	Case report	1	0	1	60
Elsayed et al. [26]	2022	UK	Case report	1	1	0	60
Fleming et al. [27]	2016	Australia	Case report	1	1	0	64
Galloway et al. [28]	2023	UK	Case series	38	27	11	59.6
Garcia-Mansilla et al. [29]	2022	Argentina	Case report	1	1	0	64
Gillis [30]	1953	UK	Case series	6	6	0	55.5
Helito et al. [31]	2014	Brazil	Case report	1	1	0	60
Kandel et al. [32]	2009	Israel	Case report	1	1	0	68
Konstantakos et al. [33]	2008	USA	Case report	1	1	0	40
Leonard et al. [34]	2010	Ireland	Case report	1	1	0	36
Li et al. [35]	2022	USA	Case series	25	22	3	57.6
Ma et al. [36]	2015	China	Case report	1	1	0	67
Mahmood et al. [37]	2020	USA	Case report	1	1	0	77
Mak et al. [38]	2008	Australia	Case report	1	1	0	84
Malagelada et al. [39]	2013	Spain	Case report	1	1	0	62
Masmoudi et al. [40]	2016	Tunisia	Case report	1	0	1	57
Maupin et al. [41]	2019	USA	Technical article	-	-	-	-
Murphy et al. [42]	2015	France	Case report	1	0	1	51
Nejat et al. [43]	2005	USA	Case series	4	1	3	61
Pasquina et al. [44]	2000	USA	Case report	1	1	0	76
Pekmezci et al. [45]	2010	USA	Case report	1	1	0	51
Perumal et al. [46]	2017	India	Case report	1	1	0	75
Prickett et al. [47]	1976	USA	Case series	2	1	1	48
Putnis et al. [48]	2020	Australia	Case report	1	1	0	56
Salai et al. [49]	2000	Israel	Case series	5	4	1	54.8
Sathappan et al. [50]	2011	Singapore	Case report	1	1	0	40
Shi et al. [51]	2014	China	Review	-	-	-	-
Sommerville et al. [52]	2006	UK	Case report	1	0	1	62
Wagner et al. [53]	2020	Argentina	Case report	1	1	0	63
Williams et al. [54]	2015	UK	Case report	1	0	1	81

### Patient summary

In total, 265 patients were identified across the included studies (Table 2). The most common cause for amputation was trauma (34.2%), with the majority trans-tibial (75.3%). The primary reason for undergoing joint replacement was osteoarthritis (77.1%), occurring most frequently on the contralateral limb (66.7%). On review of the procedures performed, 195 patients underwent total hip replacement (THR), 51 patients received total knee replacement (TKR), and 21 patients necessitated hip hemiarthroplasty; 2 patients received a combination of joint replacement, and 12 underwent bilateral procedures. The mean time to joint replacement from date of amputation was 21 years (SD 19.2) with a range from 3 weeks to 60 years. This was the same for THR and TKR, and did not change when adjusting for surgical indication.

**Table 2:** Study population demographics, including details of amputation and joint replacement. THR; Total hip replacement

	**All**		**THR**		**TKR**		**Hemi**	
**Demographics**								
Total Patients	265		195		51		21	
Male (%)	228	(86%)	163	(83.6%)	24	(47.5%)	19	(90.4%)
Female (%)	37	(14%)	26	(13.3%)	8	(15.7%)	1	(4.8%)
Unreported (%)	0	(0%)	6	(3.1%)	19	(37.3%)	1	(4.8%)
Mean Age in Years (SD)	61.1	(11.4)	58.6	(11.4)	61.0	(11.4)	67.0	(9.2)
Total Amputations	275		201		53		24	
Total Operations	279		201		56		22	
**Amputation Cause (%)**								
Trauma	94	(34.2%)	55	(27.4%)	30	(56.6%)	10	(41.7%)
Vascular	35	(12.7%)	27	(13.4%)	5	(9.4%)	3	(12.5%)
Diabetic ulcer	17	(6.2%)	17	(8.5%)	0	(0%)	0	(0%)
Osteomyelitis	15	(5.5%)	14	(7.0%)	1	(1.9%)	0	(0%)
Infection	13	(4.7%)	5	(2.5%)	8	(15.1%)	0	(0%)
Tumour	12	(4.4%)	9	(4.5%)	3	(5.7%)	0	(0%)
Congenital	12	(4.4%)	11	(5.5%)	3	(5.7%)	0	(0%)
Iatrogenic	4	(1.5%)	1	(0.5%)	3	(5.7%)	0	(0%)
Neuropathy	2	(0.7%)	2	(1.0%)	0	(0%)	0	(0%)
Idiopathic	2	(0.7%)	2	(1.0%)	0	(0%)	0	(0%)
Unreported	69	(25.1%)	58	(28.9%)	0	(0%)	11	(45.8%)
**Amputation Level (%)**								
Hindquarter	5	(1.8%)	5	(2.5%)	0	(0%)	0	(0%)
Transfemoral	49	(17.8%)	28	(13.9%)	13	(24.5%)	9	(37.5%)
Knee disarticulation	8	(2.9%)	6	(3.0%)	1	(1.9%)	1	(4.2%)
Transtibial	207	(75.3%)	158	(78.6%)	35	(66.0%)	14	(58.3%)
Ankle disarticulation	5	(1.8%)	4	(2.0%)	3	(5.7%)	0	(0%)
Foot	1	(0.4%)	0	(0%)	1	(1.9%)	0	(0%)
Unreported	0	(0%)	1	(0.5%)	0	(0%)	0	(0%)
**Indication for Joint Replacement (%)**								
Osteoarthritis	215	(77.1%)	158	(78.6%)	52	(92.9%)	5	(22.7%)
Avascular necrosis	30	(10.8%)	29	(14.4%)	0	(0%)	1	(4.5%)
Trauma (Fractured NOF)	28	(10.0%)	12	(6.0%)	0	(0%)	16	(72.7%)
Trauma (Other Fracture)	2	(0.7%)	0	(0%)	2	(3.6%)	0	(0%)
Arthrodesis	1	(0.4%)	1	(0.5%)	0	(0%)	0	(0%)
Metastases	1	(0.4%)	1	(0.5%)	0	(0%)	0	(0%)
Revision	1	(0.4%)	0	(0%)	1	(1.8%)	0	(0%)
Unreported	1	(0.4%)	0	(0%)	1	(1.8%)	0	(0%)
**Laterality of Joint Replacement (%)**								
Ipsilateral	69	(24.7%)	45	(22.4%)	14	(25.0%)	10	(45.4%)
Contralateral	186	(66.7%)	144	(71.6%)	32	(57.1%)	10	(45.4%)
Bilateral	12	(8.6%)	6	(6.0%)	5	(8.9%)	1	(9.1%)

*TKR* Total knee replacement, *Hemi* Hip hemiarthroplasty, *NOF* Neck of femur, *NB*Two patients received a combination of arthroplasty (THR and TKR) and therefore are counted in both groups [25, 35]. No sub-group demographic description of gender status in Li ADF et al., so incomplete gender demographics compared to total participants. Reporting of amputation cause and indication for arthroplasty was variable, so not all patients are accounted for. Furthermore, some patients had bilateral amputations, at differing levels, accounting for the number of total amputations recorded exceeding the number of patients

### Outcome measures

The type and frequency of outcome measures reported are summarised in Table 3. Mobility status, length of hospital stay, range of motion and patient reported outcome measures (PROMS) were the most frequently reported. All patients returned to independent ambulation post-operatively, with varying need for walking aids. The mean length of hospital stay was 10 days (range 1–33) across all subgroups of THR, TKR and hemiarthroplasty.

**Table 3:** Summary of outcome measures reported by study. PROMS; Patient reported outcome measures. ROM; Range of motion

Author	Mobility	Length of Stay	ROM	PROMS	Radiographic Follow-Up	Complications	Pain	Return to Premorbid Status	Return to Work	Revision
Amanatullah et al				*	*	*				*
Arango et al				*			*	*		
Boussakri et al	*		*		*					
Cho et al		*		*	*	*				
Cho et al		*		*		*				
Christidis et al		*						*		
Constantin et al	*	*								
Crawford et al	*	*	*	*			*			
Diamond et al	*	*		*	*		*			
Dong et al			*		*		*	*		
Dudhniwala et al	*		*	*	*					
Elsayed et al	*	*								
Fleming et al	*		*				*			
Galloway et al	*		*	*		*				*
Garcia-Mansilla et al	*	*								
Gillis	*									
Helito et al	*		*	*		*				
Kandel et al	*	*	*				*			
Konstantakos et al	*	*	*	*						
Leonard et al	*				*	*	*			
Li et al				*		*				
Ma et al	*	*	*							
Mahmood et al	*				*					
Mak et al	*		*				*			
Malagelada et al	*	*				*				
Masmoudi et al	*		*	*	*					
Maupin et al										
Murphy et al	*				*					
Nejat et al	*	*				*				
Pasquina et al	*	*			*	*				
Pekmezci et al	*						*			
Perumal et al	*	*								
Prickett et al	*		*			*			*	
Putnis et al	*		*	*		*			*	
Salai et al	*	*		*	*					
Sathappan et al	*		*	*	*					
Shi et al										
Sommerville et al	*	*	*		*	*	*			
Wagner et al	*		*							
Williams et al	*			*	*					

For patients undergoing THR, seven different PROMs were reported (Table 4): HHS (pain, function, ROM and deformity), ADL Scale (functional independence), OHS (pain and function), PROMIS-10 (health status, quality of life, pain, function), HOOS-JR (pain, function, independence, quality of life), WOMAC (pain, stiffness, physical function), PMD Scale ( pain, mobility, ambulation). The HHS was reported by eight studies, with ‘good’ (80–90) or ‘excellent’ (90–100) scores reported for the majority at long term follow-up. Meanwhile, ADL scale revealed ‘moderate’ to’full’ function achieved.

**Table 4:** PROMS reported for THR

Author	HHS	ADL Scale	OHS	PROMIS-10	HOOS-JR	WOMAC	PMD
Amanatullah DF et al	*						
Arango D et al	*						
Cho HM et al. (2018) [18]	*	*					
Cho HM et al. (2019) [19]	*	*				*	
Diamond OJ et al	*		*				
Galloway R et al			*				
Li ADF et al				*	*		
Masmoudi K et al	*						*
Salai M et al	*						
Sathappan SS et al	*						

*HHS* Harris Hip Score, *ADL* Activities of Daily Living scale, *OHS* Oxford Hip Score, *PROMIS-10* Patient-Reported Outcomes Measurement Information System, *HOOS-JR* Hip dysfunction and Osteoarthritis Outcome Score for Joint Replacement, *WOMAC* Western Ontario and McMaster Arthritis Index, *PMD* Paustel-Merle-D'Aubigné scale

For patients receiving TKR, five different PROMs were reported (Table 5): OKS (pain and function), AKSS (pain, ROM, clinical assessment, stability, function), PROMIS-10 (health status, quality of life, pain, function), KOOS-JR (pain, function, independence, quality of life), VR-12 (health status, function, mental health). The AKSS was reported by three studies, all reporting ‘excellent’ (80–100) function at final follow-up, while OKS was reported by four studies, with two studies reporting nearly normal function (40–48) and two studies reporting mild/moderate symptoms (30–39).

**Table 5:** PROMS reported for TKR

Author	OKS	AKSS	PROMIS-10	KOOS-JR	VR-12
Crawford JR et al		*			
Dudhniwala AG et al	*				
Galloway R et al	*				
Helito C et al		*			
Konstantakos et al		*			
Li ADF et al			*	*	
Putnis SE et al	*				*
Masmoudi K et al	*				

*OKS* Oxford Knee Score, *AKSS* American Knee Society Score, *PROMIS-10* Patient-Reported Outcomes Measurement Information System, *KOOS-JR* Knee dysfunction and Osteoarthritis Outcome Score for Jonit Replacement, *VR-12* Veterans RAND 12-item health survey

Cho et al. [18] performed a case–control study comparing 54 below knee amputees (BKA) undergoing contralateral THR, against 54 non-amputees matched for age, sex, weight, height and time since surgery. They found HHS (86.1; 95% CI [79–91] vs. 90.7; 95% CI [81–100]) and ADL scale (4.77; 95% CI [4-5] vs. 5.25; 95% CI [4-5]) scores were significantly lower at 3-month follow-up among amputees, compared with non-amputees. However, by 6 months this difference in functional status had resolved, with no further differences observed at final 5-year follow-up. Another study by Cho et al. (2019) retrospectively reviewed amputees and compared post-operative outcomes based upon the surgical approach used and found that where the surgeon used a posterior approach to the hip joint, HHS (80.83; 95% CI [70–96] vs. 74.51; 95% CI [64–92]) and ADL scale (3.88; 95% CI [3–5] vs. 2.45; 95% CI [2–5]) were significantly higher at 3 months [19]. However, this difference was not observed at 6 month or final 1-year follow-up.

### Complications

Of the 40 included studies, only 13 reported any post-operative complications.Where reported, complications included peri-prosthetic fracture [15, 18, 19, 35, 52], dislocation [15, 18, 19], infection (superficial and deep) [15, 35, 39, 44], aseptic loosening [15, 31], stump complications [28, 34], failed rehabilitation [43], and suboptimal implant positioning [47, 48]. Amanatullah et al. [15] reported a high overall percentage of complications (28.6%), with periprosthetic fracture (14.3%) and dislocation (8.6%) accounting for the majority.  Cho et al. [18] reported two periprosthetic fractures and one dislocation among amputees, compared with none in the non-amputee control group.  Cho et al. [19] reported a significantly higher number of falls (32.4% vs. 9.1%) among patients treated with anterolateral surgical approach in the first 3 months post-operatively, compared with those treated with the posterior approach.

### Surgical technique

For THR in amputees, the posterior surgical approach was used for 51.2%, anterolateral approach for 48.2% and direct anterior approach for 0.6%. Uncemented stems were implanted in 159 (79.1%) patients, with cemented femoral stems used in only 28 (13.9%) patients; stem implanted was not reported for 14 (7.0%). There were variations to routine surgical practice, relating to six specific techniques, mostly related to managing the residual limb during ipsilateral joint replacement. Three studies described inserting a Steinman pin into the greater trochanter, to provide rotational control and facilitate dislocation/relocation [23, 36, 53]. Four studies involved inserting 5 mm or 6.5 mm Schanz pins into the distal femur, similarly for rotational control and to allow traction for dislocation/relocation [34, 37, 45, 46]. Two studies described using bone clamps or hooks in the intertrochanteric region [16, 34], while three studies used bone forceps or clamps on the proximal femoral shaft itself, up to 5 cm distal to the lesser trochanter [17, 32, 39]. Two studies specifically mention performing additional soft tissue releases, including psoas and gluteus maximus, to facilitate femoral canal prep and achieve correct version on implantation [29, 39]. Finally, two studies reported using additional supports to maintain lateral position in lieu of a contralateral limb, with pillows secured to the bed with elastic tape, or a suction beanbag instead [42, 52].

For TKR in amputees, the medial para-patellar approach remained the standard surgical approach. However, Dong et al. [24] described placing the skin incision more medially, to avoid the tibial tuberosity and reduce potential problems associated with prosthetic loading over the scar. The reporting of implants used was poor, with a combination of cruciate retaining, posterior stabilised, highly constrained and hinged components reported [22, 24–28, 31, 47, 54]. In terms of specific surgical techniques, variations in standard practice were described for management of the residual limb and obtaining correct alignment for implants. Crawford et al. [22] described using a sterile box (polystyrene box wrapped in sterile drape) to support the knee in flexion.  Similarly, Elsayed et al. [26] utilised a sterile foam bolster, facilitating flexion/extension intra-operatively. Maupin et al. [41] reported securing the residual limb to a sterile radiolucent metal triangle with adhesive wrap, flexed with the most acute angle underneath the popliteal fossa [41]. This facilitated flexion/extension in conjunction with the standard distal transverse foot bump and lateral thigh support. Konstantakos et al. [33] created a custom prosthesis pre-operatively and sterilised the prosthetic for intra-operative use. By replicating the lower limb length and foot alignment with a prosthetic, this also facilitated the use of the standard supports, as with Maupin et al. [41]. The extra-medullary jig could therefore be used when making the tibial cuts.  Meanwhile, Putnis et al. [48] used two soft wedges of rolled towels underneath the sterile drapes to facilitate flexion. Where the residual limb is sufficiently long, however, the standard supports can be used without the need for additional equipment or novel techniques, as reported by Dudhniwala et al. [25] although Fleming et al. [27] suggest using a second assistant in this scenario.

## Discussion

The principal finding of this scoping review is that there is a lack of both observational and comparative studies on the outcome of hip and knee replacement in lower limb amputees. The existing evidence consists almost entirely of case reports and case series with significant risk of bias, amounting to low level of evidence to support any definitive conclusions or guide practice. Only one case–control study was found which compared outcomes of amputees directly to non-amputees [18], while the outcomes reported across the included studies varied widely. Therefore, it is not possible for this review to draw any definitive conclusions regarding the expected outcomes of hip or knee replacement in lower limb amputees, or to guide optimum treatment. Furthermore, as part of the primary aim of this study to broadly summarise the existing evidence base, it is important to highlight a severe lack of reporting within the literature regarding post-operative rehabilitation protocols. Given the clear challenge to ambulation faced by amputees, this is a key omission.

### Total hip replacement

From the included literature, the majority focused on THR. The mean age at surgery for amputees undergoing THR was 58.7 years; this is considerably younger than the mean age for THR among the general population of the UK, which is 69 years according to registry data [10]. Osteoarthritis was the most common indication for surgery, while THR was more frequently contralateral to the side of amputation. This evidence appears to support the hypothesis from Struyf et al. that amputation places greater strain on the remaining limb, and therefore necessitates early arthroplasty [7]. However, the time to joint replacement varied widely, with one patient only requiring surgery 60 years after bilateral amputation for congenital abnormality [25], while another patient had pre-existing hip OA exacerbated by ipsilateral amputation, leading to early surgical intervention within 2 years [39]. This clearly demonstrates there are other factors not yet accounted for which dictate the time to joint replacement from amputation, requiring further investigation.

In terms of outcome, THR is often regarded as one of the most successful surgical interventions [55]. The majority of patients report excellent, very good or good results, and 97% demonstrating improvement in function [56]. From the PROMS data reported for amputees, it appears that THR is also a highly successful procedure, with similar levels of function reported post-operatively across a range of PROMS. However, while registry data suggest 58% of THR survive 25 years before requiring revision surgery [57], it remains unclear whether the same altered biomechanics that potentially predispose amputees to OA in the first instance, would also predispose to early revision surgery.

For any patient undergoing THR, dislocation and periprosthetic fracture are devastating complications, and these appear to be uncommon among amputees. However, the low numbers in this study make definitive population wide conclusions hard to make, and one study demonstrated increased risk of falls post-operatively [19], which would predispose to both dislocation and periprosthetic fracture; this requires further study. In relation to specific complications for amputees, post-operative stump swelling was identified as a key issue by one study [34], but was less common for THR patients than TKR patients. This could limit rehabilitation by restricting ambulation through an inability to wear a prosthesis. Compression wraps and bandages could be routinely utilised in amputees to prevent this occurring.

From the available data, there was no clear preponderance for a surgical approach, with both the posterior and anterolateral approaches being used equally. However, one study found functional status among those treated with the posterior approach was significantly better at 3 months, alongside a significantly lower risk of falls [19]. However, by 6 months, these differences were no longer observed [19]. This would support the hypothesis put forward by proponents of the posterior approach, that preserving the hip abductors is important for function. Among amputees, the posterior approach would appear to optimise early functional outcomes, until those receiving anterolateral approach have had time for their abductor repair to heal and recondition. The only study to directly compare THR among amputees with non-amputees similarly found a significant difference in functional status at 3 months, resolving by 6 months [18]. This could indicate that long term functional outcomes are similar, but amputees require a longer period rehabilitation to achieve this, and the surgical approach may present a factor in this.

In terms of the intra-operative techniques, the majority of technical tips centred around the control of the residual limb to facilitate dislocation and relocation of the femoral head. No comparisons were made to suggest one technique is better than another; the utility of each is likely to be specific to the patients' individual anatomy. There was an apparent preference for uncemented femoral stems among the reported implants, with some authors concerned about cement extrusion through the distal femur in transfemoral amputees with short residual limbs [20, 23, 34, 36]. However, in cases where the distal femur is flared, with associated widening of the canal and thin cortical bone, it was recognised that cementation may provide improved stem fixation in sufficient length femurs [15, 39]. Importantly, the use of templating was highlighted by several authors as key to the pre-operative planning [20, 23, 39, 42, 50], as implant size was sometimes limited by the length of the remaining femur.

### Total knee replacement

For the studies exploring TKR, the mean age at operation was 61.0 years, similarly representing a much younger age of operation than the UK average for non-amputees, though notably older than amputees undergoing THR. The vast majority underwent surgery for osteoarthritis, more commonly on the contralateral side to their amputation. Similarly to THR, the time to arthroplasty varied widely. However, the slightly higher average age at time of operation potentially indicates a slower onset of degenerative disease at the knee compared to the hip. This warrants further investigation with clinical and biomechanical studies, to explore the comparative risk of OA in hip and knee among amputees.

In terms of outcomes, the functional status achieved was generally reported to be good, though there were no comparative studies to indicate whether the level of function is comparable to non-amputees. Overall, complications were uncommon. However, stump complications were reported more frequently among TKR patients, with both swelling and wound infection reported [28, 44]. This would appear to be a higher risk for ipsilateral TKR, as the incision may extend into the contact area for the prosthetic socket, while localised limb swelling from the surgery will be more consequential. For these patients, managing socket wear and return to ambulation are particularly challenging, with no clear consensus in the literature to support early vs delayed wear.

The technical tips for TKR similarly focused on the management of the residual limb intra-operatively. A key issue with performing TKR in the ipsilateral limb is the reduced length available for use with the bolster and support. The reporting of implants used was poor, and where available there was no clear preponderance for any type, with cruciate retaining, posterior stabilised, highly constrained and linked prostheses all being described.

### Strengths and limitations

A key strength of this review is the thorough search strategy employed, refined through consultation with an information specialist, and conformation to PRISMA reporting guidelines. As a result, the authors are confident that the studies included in this review are fully representative of the available evidence. Furthermore, this review is comprehensive in it’s inclusion of both ipsilateral and contralateral arthroplasty, facilitating a more complete summary of the population in question.

However, the scoping nature of this study inherently limits the potential for true data synthesis and comparisons. Furthermore, a formal quality assessment was not performed, in accordance with the framework set out for scoping reviews. Although the quality of available evidence was assumed to be low by the very nature of the studies included, the lack of formal quality assessment further limits the potential for discussion regarding the strength of conclusions made. When developing the protocol for this review, no formal patient and public involvement and engagement (PPIE) was sought. This may have helped guide the discussion and focus of the review in a more patient centred direction.

## Conclusion

This study has demonstrated a paucity of high quality evidence reporting on lower limb amputees undergoing hip or knee replacement, although the available evidence appears to suggest outcomes comparable to non-amputees are achievable. There is a need for more high-quality observational studies to establish the association between amputation and subsequent need for joint replacement. Furthermore, comparative studies are needed to identify whether amputees can be expected to achieve similar functional outcomes after surgery, and if they are at higher risk of complications.

### Supplementary Information

**Supplementary Material 1.**

## Data Availability

The dataset generated and analysed in this study through data extraction protocol is available from the corresponding author on reasonable request.

## Abbreviations

OA: Osteoarthritis
THR: Total hip replacement
TKR: Total knee replacement
PRISMA: Preferred Reporting Items for Systematic Reviews and Meta-analyses
MeSH: Medical Subject Heading
SD: Standard deviation
CI: Confidence interval
PROM: Patient reported outcome measure
ROM: Range of motion
BKA: Below knee amputee
HHS: Harris Hip Score
OHS: Oxford Hip Score
PROMIS-10: Patient Reported Outcomes Measurement Information System
HOOS JR: Hip Dysfunction and Osteoarthritis Outcome Score for Joint Replacement
ADL: Activities of Daily Living Scale
WOMAC: Western Ontario and McMaster Arthritis Index
PMD: Paustel-Merle-D’Aubigne Scale
AKSS: American Knee Society Score
OKS: Oxford Knee Score
KOOS-JR: Knee Dysfunction and Osteoarthritis Outcome Score for Joint Replacement
VR-12: Veterans RAND 12-iteam Health Survey
PPIE: Patient and Public Involvement and Engagement
