# Supplementary material for: Hip and knee replacement in lower limb amputees: a scoping review
Source: BMC Musculoskelet Disord. 2024 Mar 27;25:239. doi: 10.1186/s12891-024-07342-z (PMC10967077; doi:10.1186/s12891-024-07342-z)
Supplement: Supplementary file 1 — Supplementary Material 1. [file 12891_2024_7342_MOESM1_ESM.docx]

**APPENDICES**

**Appendix A**

A search strategy was developed using appropriate MeSH terms and initial scoping searches to ensure appropriate capture of relevant articles. It was refined in conjunction with an Information Specialist through PenARC search and review service. The search strategy was as follows:

1. Amputees (MeSH)

2. Amput*.ti,ab.

3. BKA.ti,ab.

4. AKA.ti,ab.

5. Limb adj2 (loss OR deficiency)

6. #1 OR #2 OR #3 OR #4 OR #5

7. arthroplasty, replacement, hip (MeSH)

8. arthroplasty, replacement, knee (MeSH)

9. Hemiarthroplasty (MeSH)

10. hip adj2 (arthroplasty OR replacement OR hemiarthroplasty).ti,ab.

11. knee adj2 (arthroplasty OR replacement).ti,ab.

12. #7 OR #8 OR #9 OR #10 OR #11

13. #6 AND #12
